# Neuroplastic Changes Following Brain Ischemia and their Contribution to Stroke Recovery: Novel Approaches in Neurorehabilitation

**DOI:** 10.3389/fncel.2017.00076

**Published:** 2017-03-16

**Authors:** Claudia Alia, Cristina Spalletti, Stefano Lai, Alessandro Panarese, Giuseppe Lamola, Federica Bertolucci, Fabio Vallone, Angelo Di Garbo, Carmelo Chisari, Silvestro Micera, Matteo Caleo

**Affiliations:** ^1^CNR Neuroscience Institute, National Research Council (CNR)Pisa, Italy; ^2^Laboratory of Biology, Scuola Normale SuperiorePisa, Italy; ^3^Translational Neural Engineering Area, The BioRobotics Institute, Scuola Superiore Sant’AnnaPontedera, Italy; ^4^Department of Neuroscience, Unit of Neurorehabilitation—University Hospital of PisaPisa, Italy; ^5^CNR Biophysics Institute, National Research Council (CNR)Pisa, Italy; ^6^Neural Computation Laboratory, Center for Neuroscience and Cognitive Systems @UniTn, Italian institute of Technology (IIT)Rovereto, Italy; ^7^Ecole Polytechnique Federale de Lausanne (EPFL), Bertarelli Foundation Chair in Translational NeuroEngineering Laboratory, Center for Neuroprosthetics and Institute of BioengineeringLausanne, Switzerland

**Keywords:** stroke, motor cortex, plasticity, callosal connections, non-invasive brain stimulation, local field potentials, rehabilitation, robotics

## Abstract

Ischemic damage to the brain triggers substantial reorganization of spared areas and pathways, which is associated with limited, spontaneous restoration of function. A better understanding of this plastic remodeling is crucial to develop more effective strategies for stroke rehabilitation. In this review article, we discuss advances in the comprehension of post-stroke network reorganization in patients and animal models. We first focus on rodent studies that have shed light on the mechanisms underlying neuronal remodeling in the perilesional area and contralesional hemisphere after motor cortex infarcts. Analysis of electrophysiological data has demonstrated brain-wide alterations in functional connectivity in both hemispheres, well beyond the infarcted area. We then illustrate the potential use of non-invasive brain stimulation (NIBS) techniques to boost recovery. We finally discuss rehabilitative protocols based on robotic devices as a tool to promote endogenous plasticity and functional restoration.

## Introduction

Following an ischemic insult within the motor cortex, one or more body parts contralateral to the infarct result impaired or paretic. The degree of the motor impairment depends on many factors, such as the extent of the infarct, the identity of the damaged region(s) and the effectiveness of the early medical care. Substantial functional recovery can occur in the first weeks after stroke, mainly due to spontaneous mechanisms (Kwakkel et al., 2004; Cramer, 2008; Darling et al., 2011; Ward, 2011; Grefkes and Fink, 2014). About 26% of stroke survivors are able to carry on everyday activities (Activity of Daily Living or ADLs, i.e., eating, drinking, walking, dressing, bathing, cooking, writing) without any help, but another 26% is forced to shelter in a nursing home (Carmichael, 2005). Impairments of upper and lower limbs are particularly disabling as they impact on the degree of independence in ADLs. Overall, a significant percentage of the patients exhibit persistent disability following ischemic attacks. Therefore, it is critical to increase our knowledge of post-stroke neuroplasticity for implementing novel rehabilitative strategies. In this review we summarize data about plastic reorganizations after injury, both in the ipsilesional and contralesional hemisphere. We also describe non-invasive brain stimulation (NIBS) techniques and robotic devices for stimulating functional recovery in humans and rodent stroke models.

## Neuroplasticity After Stroke

The term brain plasticity defines all the modifications in the organization of neural components occurring in the central nervous system during the entire life span of an individual (Sale et al., 2009). Such changes are thought to be highly involved in mechanisms of aging, adaptation to environment and learning. Moreover, neuronal plastic phenomena are likely to be at the basis of adaptive modifications in response to anatomical or functional deficit or brain damage (Nudo, 2006). Ischemic damage causes a dramatic alteration of the entire complex neural network within the affected area. It has been amply demonstrated, by many studies, that the cerebral cortex exhibits spontaneous phenomena of brain plasticity in response to damage (Gerloff et al., 2006; Nudo, 2007). The destruction of neural networks indeed stimulates a reorganization of the connections and this rewiring is highly sensitive to the experience following the damage (Stroemer et al., 1993; Li and Carmichael, 2006). Such plastic phenomena involve particularly the perilesional tissue in the injured hemisphere, but also the contralateral hemisphere, subcortical and spinal regions.

### Neuroplasticity in Perilesional Area: Map Reorganization

The most convincing evidence of post-stroke spontaneous plasticity in the perilesional area is the observation of topographical map reorganization (Harrison et al., 2013). Motor cortices show in fact a topographical organization, so that sites evoking movements of specific body parts cluster together. Maps are shaped during early life and remain quite stable in adulthood. Interestingly, they can change even in the adult by experience-dependent plasticity (such as after an intensive training) or after brain injury.

Remapping of the motor cortical areas has been observed in stroke patients via either functional Magnetic Resonance Imaging (fMRI) or Transcranial Magnetic Stimulation (TMS; Cicinelli et al., 1997, 2003; Traversa et al., 1997; Liepert et al., 1998; Rossini et al., 2001). In animal models, reorganization of motor maps has been observed using intracortical microstimulation (ICMS; Nudo and Milliken, 1996; Nishibe et al., 2010; Alia et al., 2016) or optogenetic techniques (Harrison et al., 2013).

Studies on primates have demonstrated that following an ischemic injury to the hand area of primary motor cortex (M1) there is a significant reduction of hand representation if no rehabilitative training is applied (Nudo, 2007). However, if the monkey undergoes rehabilitative exercises, the area of the hand is preserved; it is possible that training encourages reacquisition of motor skills in the impaired hand, maintaining the efficacy of corticospinal cells in driving hand motoneurons (Nudo, 2007). Other studies confirmed these results in primates and rats (Nudo, 2013; Nishibe et al., 2015; Combs et al., 2016).

Learning and post-stroke remapping seem to follow different mechanisms, even though they probably share many effectors (Krakauer, 2006; Ramanathan et al., 2006). A proof of these two different mechanisms, has been provided by Ramanathan et al. (2006) who found that complex movements evoked with ICMS do not show plasticity during learning, but they exhibit remapping during post-stroke recovery. Moreover, it has been shown that the cholinergic system plays a crucial role in remapping after stroke. In fact, Conner et al. (2005) showed that immunolesioning the cholinergic system abolished post-stroke recovery and related remapping. The cholinergic system is a component of the ascending neuromodulatory systems. Many studies reported the role of specific neuromodulators such as dopamine, norepinephrine, or serotonin in recovery from stroke also in humans (for a systematic review see Berends et al., 2009). In rodents, it has been shown that activation of modulatory neurotransmitters (via vagus nerve stimulation) in phase with motor exercise (lever pulling) improves post-stroke motor function (Hays et al., 2016).

It is well established that after a small subtotal cortical lesion, peri-infarct areas could actually vicariate lost or damaged functions (Murphy and Corbett, 2009; Dancause and Nudo, 2011). For example, following an ischemic injury in M1, premotor areas can remain functional and contribute to recovery. The ventral premotor area, which receives most of its inputs from M1, produces and releases Vascular Endothelial Growth Factor (VEGF), which has angiogenic and neuroprotective properties, in the early phase after the infarct (Nudo, 2007). In rodents, the Rostral Forelimb Area (RFA) represents a pre-motor cortex involved in the planning and execution of forelimb movements (Rouiller et al., 1993; Saiki et al., 2014; Vallone et al., 2016). The RFA shows a sustained reorganization of the motor map after stroke (Tennant et al., 2015; Touvykine et al., 2016), and preventing RFA reorganization after stroke hinders a long-lasting motor recovery even after rehabilitation (Conner et al., 2005). Consistently, inducing a second lesion in RFA after rehabilitation-induced motor recovery leads to a reappearance of the motor deficit (Okabe et al., 2016).

### Cellular and Molecular Substrates of Post-Stroke Plasticity

However, many issues regarding mechanisms underlying network reorganization and regain of motor function remain still incompletely understood. These mechanisms could involve unmasking of subthreshold pre-existing connections or sprouting of new fibers (Murphy and Corbett, 2009). In this context, the GABAergic system and the extracellular matrix could have an important role in controlling these plastic phenomena. For example, Perineuronal Nets (PNNs), specialized extracellular matrix structures made of condensed chondroitin sulfate proteoglycans (CSPGs), have been correlated with brain plasticity and repair, and preferentially surround the soma of GABAergic neurons, in particular fast-spiking parvalbumin-positive interneurons (Fawcett, 2015). The role of PNNs has been extensively investigated during the maturation of the visual system in relation to the opening and closure of the critical period (Pizzorusso et al., 2002; Deidda et al., 2015). PNNs are thought to stabilize mature connections and downregulate spine motility and functional plasticity. Following CNS injury, the degradation of PNNs, by means of injections of the bacterial enzyme chondroitinase ABC, promotes sensory-motor recovery (Bradbury et al., 2002; Soleman et al., 2012; Gherardini et al., 2015). Moreover, a recent study found a spontaneous decrease in the number of PNNs in the perilesional cortex, suggesting an enhanced plasticity (Alia et al., 2016).

The GABAergic system has also been studied in relation to the opening and closure of early “critical periods” in sensory cortices (Hensch, 2005) and in post-stroke motor recovery. Previous works showed that enhancing GABAergic signaling after stroke does not improve post-stroke performance (Madden et al., 2003), but rather induces an acute reappearance of the motor deficit in stroke patients (Lazar et al., 2010). Moreover, a correlation study in humans, showed that a reduced GABAergic inhibition is associated with functional recovery (Kim et al., 2014).

**Figure 1:**
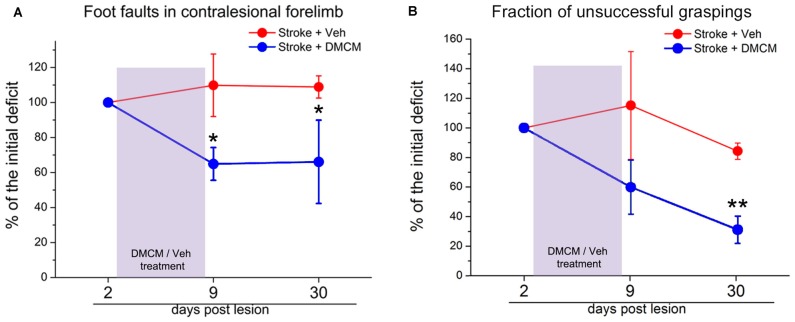
**Effect of DMCM on motor recovery after focal cortical ischemia in mice. (A)** The number of foot faults in the gridwalk task is persistently decreased after transient DMCM treatment (two way RM ANOVA followed by Tukey test, *p < 0.05; *n* = 10). **(B)** In the single-pellet retrieval task, the fraction of incorrect graspings decreases after DMCM treatment (two way RM ANOVA, followed by Tukey test, **p < 0.01). Data are shown as percentage of the initial deficit, i.e., the difference in the fraction of foot faults/incorrect graspings between day 2 and baseline (before stroke). Modified from Alia et al. (2016).

The inhibitory effect in the brain is mainly mediated by GABA signaling through a vast family of GABA_A_ receptors (Farrant and Nusser, 2005; Fritschy and Panzanelli, 2014). These ionotropic receptors are composed of different subunits and the resulting molecular assembly determines the localization in different cell districts (i.e., synaptic vs. extra-synaptic) and consequently the biological action of the receptor (phasic vs. tonic signaling; Cherubini, 2012). After a focal stroke, a substantial reorganization of these GABA_A_ receptor complexes occurs (Schiene et al., 1996). In a study from Clarkson et al. (2010), tonic GABAergic signaling appears to be increased after stroke. In fact, recordings from brain slices showed an increase in GABA_A_-receptor mediated tonic inhibition in layer 2/3 pyramidal neurons. Experimental reduction of this heightened inhibition in the first weeks post-stroke using a benzodiazepine inverse agonist, produces significant improvements of forelimb function in several behavioral tasks. Consistently, transgenic mice lacking α5- or δ-GABA_A_ receptors (mediating tonic GABA current) showed a lower functional deficit after stroke (Clarkson et al., 2010; Lake et al., 2015). In a recent study, phasic GABA was enhanced in the first week after stroke, specifically in the layer 5 of the perilesional cortex and a chronic and treatment with a positive modulator of α1-containing GABA_A_ receptors ameliorated motor outcome during the period of treatment (Hiu et al., 2016). However, these latter findings are difficult to reconcile with the clinical observation that administration of midazolam reinstates stroke deficits in hemiparetic subjects (Lazar et al., 2010). In our recent article we found a downregulation of GABAergic inhibitory presynaptic terminals in the peri-lesional area after photothrombotic stroke in mice (Alia et al., 2016). Interestingly, reducing GABA signaling in the first week post-stroke, using DMCM, an inverse agonist of GABA_A_ receptors with an high preference for α1-enriched receptors (Lüddens and Wisden, 1991; Fritschy et al., 1998), strongly improved general motor outcome, and the effects persisted well after the end of the treatment (Figure 1; Alia et al., 2016). Overall, these findings demonstrate that the GABA system offers different opportunities for therapeutic intervention and further studies are needed to better delineate the proper timing and target (phasic vs. tonic) of therapeutic treatments.

The role of excitatory neurotransmission has also been studied in relation with post-stroke recovery. Pharmacological activation of α-amino-3-hydroxy-5-methyl-4-isoxazolepropionic acid (AMPA) receptors improves motor outcome by inducing release of the neurotrophin Brain Derived Neurotrophic factor (BDNF) and phosphorylation of TrkB receptors (Clarkson et al., 2011). Moreover, increased BDNF levels and TrkB activation have been also detected after blocking NMDA receptors using memantine, a selective antagonist. As in the previous study, activation of the BDNF signaling pathway was associated with an improved motor performance and increased area of forepaw sensory maps (López-Valdés et al., 2014). Thus, the activity-dependent release of BDNF appears to be essential for the motor recovery (Berretta et al., 2014). In fact, BDNF modulation has also been suggested to mediate the therapeutic effect of treatments increasing perilesional cortex excitability, such as transcranial direct current stimulation (tDCS; Clarkson and Carmichael, 2009; Fritsch et al., 2010).

Stroke induces the production of various inhibitors of neural regeneration, sprouting and plasticity, such as myelin components (Nogo-A, myelin-associated glycoprotein), and guidance molecules (ephrins, semaphorins). The application of drugs able to neutralize the effect of anti-plastic agents, such as Nogo-A antibodies has been seen to encourage axon regeneration, sprouting and functional recovery in a variety of animal models of cortical and spinal injuries (Freund et al., 2006; García-Alías et al., 2009; Maier et al., 2009; Alilain et al., 2011). Particularly, anti-Nogo-A antibodies treatment delivered before motor training strongly improve motor recovery (Wahl and Schwab, 2014). In this study, timing of the treatments was found to be critical, indeed delivering anti-Nogo-A during motor training is not effective to improve motor deficits (Wahl and Schwab, 2014).

After stroke, it has been reported a consistent change in terms of “sprouting markers”, with an increase of classical axonal growth markers such as, growth-associated protein of 43 KDa (GAP43), CAP23, c-Jun, in the peri-infarct region but also a parallel increase of growth inhibitory genes such as ephrin-A5, CSPGs and others, at later time points (Carmichael et al., 2001, 2005; Caleo, 2015).

### Post-Stroke Changes in Contralateral Hemisphere and Interhemispheric Coupling

A critical point in literature about stroke evolution and functional recovery is the role of the uninjured hemisphere. It is well established both from animal and patient studies that contralesional neuronal connections appear to be altered as a result of a unilateral cortical damage (Jones, 1999; Witte et al., 2000; Papadopoulos et al., 2006; Jones and Jefferson, 2011; Dancause et al., 2015). Human functional imaging studies on post-stroke patients, using PET and functional MRI (fMRI), have identified a role of the healthy hemisphere in recovery. An enhanced activity in the contralesional hemisphere has been reported in patients in the first 10 days post injury, followed by an increase in the ipsilesional one (3–6 months). This sequential activation was related to improvements in motor performance (Marshall et al., 2000; Ward et al., 2003). In preclinical studies on rodent models, the activity of the contralesional hemisphere was found enhanced in the very acute stage after stroke, when the deficit was more pronounced, and was followed by perilesional activation at later stages during the recovery phase (Dijkhuizen et al., 2001). Focal stroke targeted in the somatosensory cortex (SSC) induced a transient but consistent increase of basal metabolism and field potentials in the healthy hemisphere, in terms of baseline activity and response-related activity after somatosensory stimulation of the unaffected forelimb (Takatsuru et al., 2009). Other preclinical studies in SSC showed lesion-induced changes in the contralesional cortex sensory map, with an increase in dendritic branches of layer V pyramidal cells. These changes appear to be increased if the animal is subjected to early exercises that enhance motor skills (Biernaskie et al., 2004; Gonzalez et al., 2004). Moreover, two-photon imaging *in vivo* studies have highlighted phenomena of structural rearrangements of neurons in the healthy hemisphere, both at the level of individual cells and of whole circuits. In particular, data have shown a transient, localized increase in the turn-over of dendritic fungiform (mushroom) spines, which are usually known to be highly stable in a healthy brain, in a time period limited to 1 week post-stroke. Overall, the number of dendritic spines remained unchanged probably because of a balanced number of new-born and degenerated contacts (Takatsuru et al., 2009). This could be a primary difference in mechanisms of post-stroke modifications between the intact and the injured hemisphere. Indeed in the ipsilesional cortex a net gain of dendritic spines has been observed (Brown et al., 2007). The loss of stability of dendritic spines in the healthy hemisphere can be explained by increased baseline and sensory input from the periphery (Takatsuru et al., 2009). Finally, experimental silencing of the healthy hemisphere with the GABA_A_ agonist muscimol within hours after stroke can improve functional recovery and the duration of the inactivation is directly correlated with improvement (Mansoori et al., 2014).

These results indicate an involvement of the healthy hemisphere in functional alterations following a unilateral ischemic injury. However, whether the healthy hemisphere has a positive or negative impact on recovery is still controversial (Murase et al., 2004; Hummel et al., 2008; Di Pino et al., 2014). In fact, there are many evidences that in some cases the activity of the healthy hemisphere can worsen motor recovery. For example, a recent quantitative electroencephalography (EEG) study in stroke survivors showed that the increase of contralesional hemisphere activity, during the acute phase, is related to negative final outcome. In fact, the increase of the contralesional power associates to an interhemispheric communication breakdown (Assenza et al., 2013). A possible hypothesis is that the lesion volume and the amount of spared tissue in the injured hemisphere could influence the role of the healthy cortex. In particular, when the lesion is sufficiently small to allow the reorganization of spared adjacent motor areas, the contralesional hemisphere activity would have a negative impact on the recovery. Conversely, when the lesion extent is so large to involve most of motor areas, the healthy hemisphere could be important to vicariate lost functions (Di Pino et al., 2014). In line with this theory, acute pharmacological inactivation of the healthy hemisphere (via lidocaine injection), induced different effects in ischemic rats, depending on the lesion size. Animals with large lesions were dramatically affected by lidocaine administration and showed a strongly impaired performance in a reaching task (Biernaskie et al., 2005).

Changes in the interaction between the two brain hemispheres after stroke are also a widely investigated topic. The neural activity in the brain motor areas is functionally coupled between the two hemispheres (Kinsbourne and McMurray, 1974; Vallone et al., 2016) and the lateralization of neural activity during movements is likely to be related to interhemispheric inhibition between motor areas exerted via transcallosal connections (Bütefisch et al., 2008). After a cortical injury, the subjects recovering from stroke showed changes in these interhemispheric influences (Kobayashi and Pascual-Leone, 2003; Mohajerani et al., 2011) which are thought to be caused by an imbalance in the mutual interhemispheric inhibition between the two motor cortices (Murase et al., 2004; Vallone et al., 2016) that could be an obstacle for motor recovery.

It has been proposed that after unilateral stroke, transcallosal connections could transmit an excessive inter-hemispheric inhibition onto the unaffected hemisphere. Despite the scarce knowledge about the mechanisms mediating these phenomena, the involvement of transcallosal glutamatergic connections acting on pyramidal tract neurons via local GABAergic interneurons is widely accepted (Reis et al., 2008). The role of the corpus callosum in inhibitory interhemispheric mechanisms have been strongly demonstrated through transcranial stimulation studies on patients with agenesis of the callosum (Meyer et al., 1995). FMRI studies have shown an increased bihemispheric activation during movements of the affected limb in early post-stroke patients (Loubinoux et al., 2003) and suggest persistent alterations in intracortical and transcallosal connections, despite a good degree of functional recovery of patients (Nair et al., 2007). This is probably due to a decrease of the ipsilesional neuronal activity and an increase of the contralesional one (Murase et al., 2004; Fregni and Pascual-Leone, 2007), so that the imbalanced activation of the healthy hemisphere causes an increased inhibitory transcallosal signal to the affected side. In such a scenario, low-frequency inhibitory repetitive TMS (rTMS) could be applied over the intact side as a therapeutic strategy to increase rehabilitation-induced motor performances (Nowak et al., 2009; Kim et al., 2010; Silasi and Murphy, 2014; Caleo, 2015). This is consistent with the theory that a balanced, mutual inhibition exists between the two hemispheres and that a unilateral lesion can destroy this equilibrium with the healthy hemisphere taking control and interfering with the activity of the perilesional, spared tissue (Murase et al., 2004; Vallone et al., 2016). Evidences from animal models suggest that experimental inactivation of contralesional hemisphere could actually increase motor recovery, especially when the treatment is prolonged (Barry et al., 2014; Mansoori et al., 2014; Dancause et al., 2015). In humans, application of inhibitory rTMS to the non-lesioned hemisphere improved paretic hand reach-to-grasp performance, movement time and coordination (Tretriluxana et al., 2013). However, other studies have failed to support this idea and the role of the healthy hemisphere in post-stroke recovery remains controversial (see below). Thus, the significance of contralesional activation during execution of a motor task with the affected limb is still uncertain: it could represent an epiphenomenon of recovery, an adaptive neuroplastic process or even a sign of maladaptive modifications that might interfere with the recovery process.

## Studies of Functional Connectivity: Inter-Hemispheric Breakdown in Stroke

### Computational Analyses of Post-Stroke Functional Connectivity

Recently, thanks to the advancement of new technology and theoretical/computational analyses, researches on stroke have focused on the effects produced on distant brain areas by a local ischemic injury (see for detailed reviews, Grefkes and Fink, 2011, 2014; Carter et al., 2012; Silasi and Murphy, 2014). This type of approach has been called connectivity-based and is related to the concept of connectome, which is defined by the connections between neurons (Sporns et al., 2005). Three major different spatial scales can be considered in studies of the connectome: microscopic (synapses), mesoscopic (regional interactions, for instance connections from homotopic brain areas) and macroscopic (e.g., thalamus-cortex interactions). The human brain is an extremely complex system containing a huge number of neurons (on the order of 10^11^) highly and specifically interconnected (a neuron typically receives 10^4^ inputs from other cells) and nowadays a precise connectome is missing (see Silasi and Murphy, 2014 and references therein). The development of new theoretical and computational tools for the analysis and modeling of neural signals can be an important step forward to dissect the structure of neural circuits. As an example, in the framework of the statistical data assimilation problem (Abarbanel, 2013), we should ask ourselves which kind of experimental data we need to infer the exact geometry of a large neural network, composed by thousands of interacting neurons. Usually, due to the sparseness of data, this missing information is replaced by several assumptions that can be crucial in building such large scale network models.

Mainly due to these limitations, in many studies on human stroke, the definition of connected brain areas is based on the estimation of the interdependence level (and directional prevalence of the coupling) between the neural signals recorded in the corresponding regions. To get this, specific measures quantification, borrowed from methods of linear and nonlinear time series analysis, can be employed. Well known examples of these measures are: the cross-correlation, the mutual information, the Granger causality, the transfer entropies (Abarbanel, 1996; Kantz and Schreiber, 2004; Pereda et al., 2005) and Dynamic Causal Modeling (DCM; Friston et al., 2013).

These signal processing techniques aim at finding common dynamical indices to be used as indicators of functional coupling between different areas (e.g., intact and injured motor cortex) in humans and animal models of stroke. Longitudinal variations in the values of such indicators can be employed to quantify the level of synaptic reorganization and plasticity during the rehabilitation process, medical therapy and for identifying suitable prognostic indicators.

From a theoretical perspective, the purpose of these studies is to understand how the dynamics of coupled neural networks (brain areas) is modulated by the local modifications of inhibitory and excitatory neurotransmissions in one of them (here represented by the ischemic area). This system level view could provide additional insight into basic fundamental questions about the mechanism of functional segregation and integration in the nervous system (Tononi et al., 1994) and neuroplastic phenomena (see Silasi and Murphy, 2014 and references therein).

In human studies of stroke, the typical techniques suitable to record the neural activity of the brain are fMRI (Logothetis, 2008; James et al., 2009), EEG and Magnetoencephalography (MEG; see for detailed review Grefkes and Fink, 2011, 2014; Carter et al., 2012).

All of the above techniques have the advantage to be non-invasive and therefore suitable for studies on human subjects. However they are difficult to relay to the underlying neural activity (Buzsáki et al., 2012, see for fMRI signals Logothetis, 2008). For example, the direct relationship between neuronal activity and EEG recordings it is not well defined because the electrical activity, generated by thousands of neurons, must diffuse through various media (cerebrospinal fluid, dura mater, cranium, muscle and skin) that distort and attenuate the signals.

To avoid distortion effects, intra-cortical recordings called Local Field Potentials (LFPs) can be employed in animal models. LFP signals represent the low frequency part (<500 Hz) of the extracellular potential generated by the flow of trasmembrane currents in neuronal populations located near the recording electrode (Buzsáki et al., 2012). In contrast to the high-frequency part (which reflects spiking activities), the LFP carries information on the collective synaptic activity of thousands of interconnected neurons. Therefore, the interpretation of the LFP is challenging, and even knowing that the main contribution to the LFP recording is due to the synaptic currents (since they are slow events that can be more easily synchronized than fast events), several factors such as fast (Na^+^) action potentials, calcium spikes, spikes after hyperpolarization, gap junctions, neuron-glia interactions and ephaptic effects can influence to the LFP signal. Nevertheless, it is believed that LFP recordings are the most informative signals of the underlying neural activity generated by an ensemble of coupled neurons (Buzsáki et al., 2012).

### Changes in Interhemispheric Interactions After Stroke

Recent studies (see below) demonstrated that connectivity measures can improve our ability to correlate behavioral deficits to clinical indices of dysfunction (both structural and functional). In this context, many studies have investigated the interactions between brain hemispheres before and after stroke.

In clinical studies, using MEG signals recorded from stroke patients in resting state condition, changes in alpha-band functional connectivity, both in the peri-lesional and contra-lesional cortex, have been related to improvements of functional outcomes of upper extremities (Westlake et al., 2012). Moreover, previous fMRI data have demonstrated a correlation between post-stroke loss of sensorimotor function and deterioration of inter-hemispheric functional connectivity in animals (van Meer et al., 2010). In humans, inter-hemispheric interactions have been investigated mainly by resting state fMRI signals (Grefkes and Fink, 2011, 2014; Carter et al., 2012) reporting loss of coherence between inter-hemispheric communication that predicts post-stroke behavioral deficits. Accordingly, the results obtained by analyzing EEG signals from stroke patients support the idea that a reduction of the inter-hemispheric coupling is correlated to functional deficit and a promising target for rehabilitation is the restoring of the inter-hemispheric communication (Wu et al., 2011; Assenza et al., 2013; Finnigan and van Putten, 2013 and references therein).

In a recent report (Vallone et al., 2016), our group employed a photothrombotic mouse model of stroke in caudal forelimb area (CFA) to study the plastic reorganization in the spared circuits of the adjacent premotor area (RFA). Our aim was to understand the potential role of RFAs in recovery of forelimb function after an ischemic episode in CFA (Rouiller et al., 1993; Guggenmos et al., 2013).

**Figure 2:**
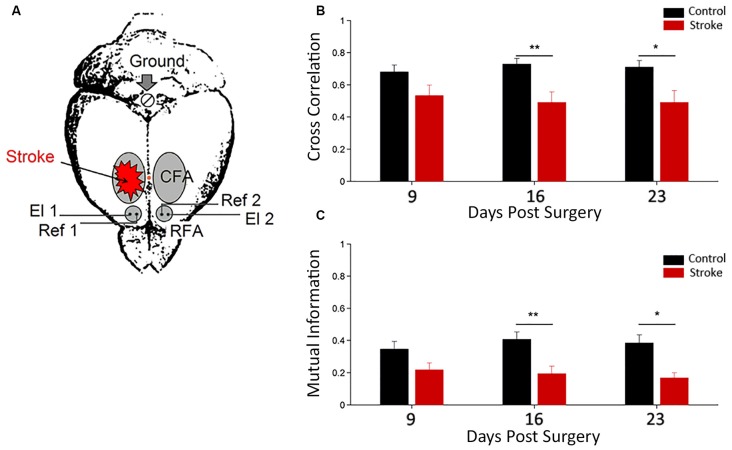
**Functional interhemispheric coupling in stroke mice. (A)** Schematic drawing of stroke and electrode location for local field potential (LFP) recordings. Unilateral phototrombotic stroke was induced in caudal forelimb area (CFA) and bipolar recording (El1 and El2) and reference (Ref1 and Ref2) electrodes were inserted in both rostral forelimb areas (RFAs). A surgical screw (Ground) was placed in the occipital bone and used as ground reference. Orange dot represents Bregma position. Cross correlation **(B)** and Mutual Information **(C)** measures between the two hemispheres in control (black) and ischemic (red) animals are shown. Data are means ± standard errors. Modified from Vallone et al. (2016). *P < 0.05, **P < 0.01.

To investigate the electrophysiological changes within and among Pre-Motor Areas, LFPs were recorded from both RFAs in freely moving mice after a cortical lesion in CFA (i.e., 9–16–23 days after surgery, see Figure 2A). These time intervals were chosen since they roughly correspond to highest period of circuit plasticity in humans (i.e., 3 months after stroke, see Zeiler and Krakauer, 2013). Quantitative methods of time series analysis were used to assess longitudinal changes in electrical neural activity (Vallone et al., 2016).

Cross correlation and mutual information analyses were employed as linear and nonlinear measures of functional coupling between the two hemispheres, respectively (Figures 2B,C). We found a dampening of the functional connectivity between RFAs in ischemic animals (with respect to control group) at day 16 and 23 after surgery (Vallone et al., 2016). These data are consistent with resting-state fMRI in humans (Grefkes and Fink, 2011, 2014; Carter et al., 2012) and rats (van Meer et al., 2010). These studies reported a significant loss of functional connectivity between the two hemispheres following stroke that slowly recovered in parallel with spontaneous behavioral improvements.

Furthermore, in this study (Vallone et al., 2016), a significant stroke-dependent reduction in inter-hemispheric cross-correlation and mutual information for gamma band (30–50 Hz) at day 9 after surgery was observed. The values of the mutual information for delta band (0.5–4 Hz) also indicated a reduction in stroke mice at day 9. Thus, changes in inter-hemispheric coupling for the gamma and delta range preceded the variations observed using the whole LFP signal. Interestingly, our results confirm a key role of gamma oscillations in cross-talking between brain regions (Buzsáki and Schomburg, 2015), especially in combination with slower frequencies (Lisman and Jensen, 2013; Buzsáki and Schomburg, 2015) and support the idea that stroke impairs brain functions because it disrupts communication in large brain networks (see Carter et al., 2012).

Using DCM on fMRI signals (see Friston et al., 2013 and references therein), stroke patients have shown a reduced excitatory influence from the contra-lesional to the ipsi-lesional PMAs and also inhibitory effects are significantly reduced from the ipsi-lesional to contra-lesional M1 cortices (see Grefkes and Fink, 2014 and references therein). Notably, in stroke patients the inter-hemispheric coupling parameters increased with recovery predicting better functional outcome after 3–6 months (see Grefkes and Fink, 2014 and references therein).

## Non-Invasive Modulation of Post-Stroke Plasticity in Humans

Thanks to neuroplasticity, the CNS compensates for the functional impairment after stroke. In particular, adaptive plasticity allows the acquisition of new skills, learning, memory, adaptation to new environments throughout the life span (Rossi et al., 1998; Hosp and Luft, 2011). Moreover in the last decade it has been described a different type of neural plasticity due to the injury and to excessive training (Quartarone et al., 2006), named “maladaptive plasticity”. Clinically, this phenomenon is relevant in several functions such as the vicariation of the upper limb movements with compensatory or substitutive movements and the delayed onset involuntary abnormal movements (Takeuchi and Izumi, 2012). Furthermore, several studies have reported that maladaptive plasticity weakens motor function and limits motor recovery after stroke (Murase et al., 2004; Rijntjes, 2006; Takeuchi et al., 2007). Moreover, it is thought to contribute to the pathogenesis of phantom pain and dystonia (Quartarone et al., 2006; Flor, 2008).

The idea to stimulate plasticity in the injured CNS to improve motor recovery is referred as “top-down” approach, and comprises the use of promising tools like plasticizing drugs or NIBS techniques (Chisari, 2015).

NIBS paradigms are applied in human stroke subjects in various ways (Dayan and Cohen, 2011; Dayan et al., 2013; Sandrini and Cohen, 2013; Wessel et al., 2015), both in experimental evaluative protocols and for therapeutic applications (Ziemann et al., 1996; Huang et al., 2005; Hummel and Cohen, 2006; Reis et al., 2008) as a possible technical adjuvant to customarily used neurorehabilitative treatments to enhance motor recovery (Liew et al., 2014; Chisari et al., 2015).

TMS is a NIBS technique that allows to study and to modulate the cortical excitability. The biophysical mechanisms induced by magnetic stimulation are still not completely understood. Given that the axons are the most effective conductors in the CNS, for their higher density of ion channels, the prevailing hypothesis is that they are preferentially affected by the TMS pulse, which may activate both inhibitory and excitatory neurons (Huerta and Volpe, 2009).

The use of particular protocols of rTMS enabled to produce a prolonged modification in cortical excitability with long-term potentiation (LTP)—and long-term depression (LTD)—like changes. In the motor system, low-frequency (1 Hz) rTMS inhibits cortical excitability, creating a transient “virtual lesion” (Chen et al., 1997). Instead, high-frequency (5–20 Hz) rTMS produces an increase in cortical excitability (Pascual-Leone et al., 1994), which can facilitate learning of motor sequences (Kim et al., 2004), though the effects may vary (Agostino et al., 2007).

A way to induce longer-lasting effects than conventional rTMS paradigms (Dayan et al., 2013) is theta-burst stimulation (TBS), which involves the application of a burst of three 50-Hz pulses in trains repeated at 200-ms intervals. Continuous TBS (cTBS) consists of the application of burst trains for 20–40 s and has an inhibitory effect on corticospinal excitability. Instead, for intermittent TBS (iTBS), burst trains with a duration of 2 s are applied over a total of 190 s, with the trains repeating every 10 s (Huang et al., 2005). iTBS can induce LTP-like changes in the stimulated hemisphere and LTD-like changes in the opposite hemisphere.

Another stimulation protocol widely used for demonstrating LTP-like and LTD-like phenomena is paired associative stimulation (PAS). PAS takes advantage of the principles of associative plasticity by repeatedly coupling a low-frequency peripheral stimulation from the median nerve with a cortical TMS pulse applied over contralateral motor cortex, with an inter-stimulus interval (ISI) of 10–25 ms (Stefan et al., 2000). An ISI of 10 ms induces a depression of TMS-evoked MEPs, while enhancement of cortical excitability is consequent to the use of 25 ms of ISI, with effects of at least 1 h of duration and resembling LTP-like and LTD-like mechanisms. Protocols using PAS are particularly relevant because they demonstrate some characteristics of spike timing-dependent plasticity (Wolters et al., 2003): the order and precise temporal interval between presynaptic and postsynaptic spikes determine the sign and magnitude of LTP-like or LTD-like synaptic changes.

Transcranial electric stimulation (tES) is a method that has attracted significant attention because its application is thought to induce neuromodulation, as shown by improvements in behavioral and cognitive performance in normal and pathological subjects (Miniussi and Vallar, 2011). Different types of tES are differentiated by specific modalities of current discharge (e.g., direct vs. alternating) that might have different neuromodulatory effects on cortical networks.

Among tES techniques, tDCS offers the possibility to change cortical excitability in a polarity-specific manner (anodal vs. cathodal; Nitsche and Paulus, 2000) through the application of electrodes with different polarity to different locations on the surface of the skull to excite the underlying neural tissue (Utz et al., 2010). tDCS effects are most likely induced by membrane polarization, altering the firing rates of neurons (Fritsch et al., 2010). Anodal tDCS induces depolarization, while cathodal tDCS induces hyperpolarization, so that anodal stimulation produces excitation and cathodal stimulation produces inhibition (Liebetanz et al., 2002).

In part, the use of NIBS techniques is based on the interhemispheric competition model, based on the concept that motor deficits in stroke patients relate to reduced output from the affected hemisphere and excessive interhemispheric inhibition from the unaffected hemisphere to the affected hemisphere (Kinsbourne, 1977, 1980; Murase et al., 2004; Takeuchi et al., 2005). Therefore, improvement in motor deficits can be achieved by increasing the excitability of the affected hemisphere or decreasing the excitability of the unaffected hemisphere (Ward and Cohen, 2004; Nowak et al., 2008). This model has been recently brought into question by Di Lazzaro et al. (2013): they used inhibitory TBS of affected hemisphere in chronic stroke patients to verify if this intervention had the potential to enhance recovery, possibly via a homeostatic increase in learning capacity. Results showed clinical improvements for up to 3 months post-treatment, suggesting the possibility to design protocols of inhibition of affected hemisphere for chronic stroke patients. It is conceivable that either upregulation or downregulation of activity in the affected hemisphere may promote recovery depending on different factors like magnitude of baseline motor function (Fridman et al., 2004) as highlighted in a protocol using iTBS followed by rehabilitative motor training (Volz et al., 2016).

Excitability enhancement in the motor cortex appears to be required for motor learning (Pascual-Leone et al., 1998; Muellbacher et al., 2002; Reis et al., 2009; Censor et al., 2010; Schambra et al., 2011). Therefore, NIBS can facilitate motor learning and induce motor recovery by directly or indirectly increasing the excitability in the ipsilesional motor cortex. In fact, compared to motor training or rTMS alone, pairing motor training with rTMS results in prolonged performance improvements and functional neural plasticity in the ipsilesional motor cortex (Nowak et al., 2008; Takeuchi et al., 2009).

NIBS-induced metabolic changes may also promote neural plasticity and motor recovery after stroke (Conchou et al., 2009). Furthermore, excitatory NIBS over the affected hemisphere can induce LTP-like changes in the affected hemisphere and promote motor recovery after stroke (Di Lazzaro et al., 2010). Therefore, NIBS may resolve impairment of experience-dependent plasticity in the affected hemisphere after stroke (Carmichael, 2006; Di Filippo et al., 2008; Takeuchi and Izumi, 2012). In addition fMRI and EEG studies proved that NIBS is able to modulate neural networks also in brain regions far from the stimulated area (Grefkes et al., 2010; Takeuchi et al., 2010). Excitatory rTMS over the affected hemisphere has been shown to reduce neural activity in the contralesional motor cortex, in addition to facilitation of the ipsilesional motor cortex (Ameli et al., 2009). Moreover, inhibitory rTMS over the unaffected hemisphere reduced the connectivity of both hemispheres and enhanced coupling between the primary and non-primary motor cortices in the affected hemisphere (Grefkes et al., 2010; Takeuchi et al., 2010). Enhanced excitability in the unaffected hemisphere inhibits the affected hemisphere via excessive interhemispheric inhibition and weakens motor function of the paretic side (Murase et al., 2004). Although the change in neural coupling after excitatory NIBS remains still unclear, normalized excitability of both hemispheres and reconstruction of effective connectivity between the primary and non-primary motor cortices in the affected hemisphere after NIBS may contribute to motor recovery in stroke patients (Takeuchi and Izumi, 2012).

The pattern of neural network activation in both hemispheres has important influences on the effect of NIBS therapy for stroke patients (Nowak et al., 2008; Ameli et al., 2009). Therefore, it seems to be important to develop predictors of NIBS response. In fact the neural impact of a NIBS therapy is not determined only by the properties of the stimulus but also on the activation state of the brain. This “state-dependency” is a general feature of cortical neural processing and it plays an important role on the efficacy of TMS protocols (Silvanto and Pascual-Leone, 2008). To address these issues, more recently EEG combined with TMS has open the new chapter of “closed-loop NIBS” (Raco et al., 2016), which with millisecond precision enables selective interference with ongoing brain activity with high temporal, spatial and spectral precision. This approach has the important advantage to take into account not only inter- individual differences in the excitability and connectivity of brain networks but also the time-course of dynamic changes of network reorganization during stroke rehabilitation. Zrenner et al. (2016) argued that two different closed-loop interactions can be differentiated: a “brain-state dynamics” loop, used to couple with and modulate the trajectory of neuronal activity patterns, and a “task dynamics” loop, that is the bidirectional motor-sensory interaction between brain and (simulated) environment, and which enables goal-directed behavioral tasks to be incorporated. Both loops need to be considered and combined to realize the full experimental and therapeutic potential of closed-loop neuroscience and to interactively optimize neuromodulatory efficacy.

## Robot-Assisted Rehabilitation Following Stroke

### Features and Advantages of Robotic Devices

The use of robotic devices aimed at improving the recovery of upper limb motor function in post-stroke therapy was first introduced in the 1990s with the MIT-Manus system, a mechanized device to assist planar reaching movements (Aisen et al., 1997), and with the ARM Guide, a robotic device to assist reaching movements in a range of directions in space, but restricted to follow a linear trajectory (Reinkensmeyer et al., 2000). Lum and colleagues (Burgar et al., 2000; Lum et al., 2002) have then demonstrated the efficacy of robot-assisted whole arm exercises in 3D with the Mirror Image Movement Enabler (MIME). Since then, several other studies have been carried out with the common goal to design and control robotic devices able to monitor and administer exercises to the patient, by eliciting motor brain plasticity and therefore improving motor recovery (Amirabdollahian et al., 2003; Reinkensmeyer et al., 2004; Micera et al., 2005; Schmidt et al., 2005).

Devices for upper limb rehabilitation can be broadly classified into three types, based on the different type of motion assistance they can provide: *active devices* that provide an active motion assistance and need at least one actuator, able to produce movement of the upper-extremity along a defined trajectory; *passive devices* that offer non-powered support of the limb during movement attempts (elastic bands or springs); and *interactive devices* that combine actuators and control strategies allowing for the correction of “wrong” motor exercise but also for the modification of the control parameters based on ongoing participant performance during the training (Marchal-Crespo and Reinkensmeyer, 2009).

A further categorization of robotic devices for upper limb motor rehabilitation can be based on their mechanical structure: *end-effector-based* and *exoskeleton-based* systems (Maciejasz et al., 2014). In end-effector-based devices, only the most distal part of the robot (i.e., end effector) is attached to patient’s upper limb extremity (hand or wrist). Movements of the end effector change the position of the upper-limb extremity, but also indirectly affect the position of the other segments of the patient’s upper-limb. Exoskeleton-based systems have a more complex mechanical structure that mimics the structure of patient’s limb. They allow for independent and concurrent control of many robot joints, which directly affect the position of correspondent joints of patient’s arm. The mechanical and control algorithm complexity of such devices is usually higher than the end-effector-based devices, because of the need to adjust lengths of particular device’s segments to the lengths of the patient arm’s segments or to manage the high number of movements (high number of degrees of freedom, DOF) allowed. The number of DOF, i.e., all independent movements (i.e., displacements or rotations) that can be performed in all the joints of a robotic device, generally depends on the target of the rehabilitation process: for example, the devices for the rehabilitation of the whole upper limb can have up to 10 DOF (Ren et al., 2009) whereas hand exoskeleton can reach even 11 DOF (Hasegawa et al., 2008). However many devices with a limited number of DOF have been developed. Planar robots have only 3 DOF, 2 translational and 1 rotational, allowing movements only on a specified plane (Aisen et al., 1997; Colombo et al., 2005). Planar devices reduce the range of upper limb movements that can be trained, but also cuts down the cost of the system, leading to an improved cost-effectiveness of robot assisted therapy (Wagner et al., 2011). However, when the working plane is appropriately selected, this range of training motion may be sufficient in most of therapeutic scenarios (Maciejasz et al., 2014).

Robotic systems have many properties, as high repeatability, possibility to perform a great amount of exercises in a single session and high intensity of task-oriented training (Posteraro et al., 2009). Many of these devices can be also adapted for the needs of the patients, allowing for a customization of the therapy. For example, exoskeleton-based systems can be tailored based on the length of the patient arm’s segments whereas end-effector-based devices can provide different types of motor exercises in agreement with the spared motor patient’s ability (Maciejasz et al., 2014). Furthermore, recent studies have also attempted to improve motivation in stroke rehabilitation using these robotic devices coupled with elements of virtual reality (e.g., audiovisual elements, score displays and cognitive challenges; Novak et al., 2014). Indeed, motivation could promote exercising for longer periods, increasing the total amount of training?

These devices incorporate several sensory components (e.g., encoders, accelerometers, load cells, etc.) allowing for a complete feedback and monitoring of the therapy progress over the time, even adjusting the degree of robot assistance based on the progress of the patient. Thus, it is possible to obtain an evaluation of patient motor performance that is extremely accurate and objective (Hidler et al., 2005; Prange et al., 2006). Several kinematic and kinetic measures can be recorded offering a complementary evaluation of the motor performance with respect to traditional methods, i.e., clinical scales (Bosecker et al., 2010). A number of parameters have been defined quantifying, for example, the smoothness of the movement, the mean speed, the movement accuracy, the mean arrest period and also the details of the constituent sub-movements (Rohrer et al., 2002; Rohrer and Hogan, 2006; Panarese et al., 2012, 2016). Upper limb weakness (i.e., lack of strength) is another common consequence of stroke and its time evolution is an important clinical parameter. Robotic systems can continuously monitor the force signal exerted by the patient by means of force sensors (i.e., pressure sensors or load cells) and extract important parameters as average force amplitude, direction and number of force peaks performed during the motor task (Reinkensmeyer et al., 2000; Colombo et al., 2010).

### Clinical Aspects of Robot-Mediated Motor Recovery in Humans

Up to now, a wide range of strategies and devices have been developed for promoting motor recovery after stroke by taking advantage from the brain’s ability to reorganize its neural networks after the injury (Lamola et al., 2014). Traditional approaches towards rehabilitation can be qualified as “bottom-up” approaches as they operate at the peripheral effectors and expect for central nervous modifications (Chisari, 2015). Currently, robotic technologies and mechatronic devices represent the modern version of bottom-up treatments providing a high dosage of task-oriented training to patients affected by different degree of functional impairment (Fasoli et al., 2004). Robotic training can increase the intensity of therapy, and bring down the requirement of assistance for rehabilitation, with a consequent decrease of costs for the health care system (Barbeau and Visintin, 2003), especially in case of gait impairment.

Robotic systems for gait recovery can be essentially divided in two main categories: end-effectors or electromechanical exoskeletons. Examples of end-effector devices are the “G-EO-System” (Hesse et al., 2010), the “Lokohelp” (Freivogel et al., 2009) and the “Gait Trainer GT 1” (Hesse et al., 2010). End-effector systems are characterized by the absence of any constraint at the hip and knee joints and the presence of foot platforms which movement simulates the phases of the gait cycle (Hesse et al., 2010). Among the exoskeleton systems we find the “LOPES” (Veneman et al., 2005) and the “Lokomat” (Colombo et al., 2000). This kind of devices can be considered as “fixed” robotic gait orthosis that move the patient’s lower limbs miming the gait kinematics by acting at hip and knee level (Hesse et al., 2010). The comparison of end-effector and exoskeleton devices in a systematic review (Mehrholz and Pohl, 2012) suggested that post-stroke walking recovery may depend on the type of robotic device, even if the lack or a direct comparison between the two typologies of device make difficult to base a final clinical recommendation.

Although a set of data does not support a clear benefit of robotic gait training when compared with therapist-assisted one (Hidler et al., 2005; Hornby et al., 2008) and the matter is still disputed (Husemann et al., 2007), several publications (Colombo et al., 2001; Werner et al., 2002; Schwartz et al., 2009; Westlake and Patten, 2009) highlighted that robotic gait training is at least equivalent to therapist-assisted treadmill training in terms of efficacy and that electromechanical driven gait rehabilitation leads to a more symmetric gait pattern, to a lower spasticity and a more physiologic gait kinematics (Mayr et al., 2007). Nevertheless, despite the fact that robotic gait rehabilitation is paving the way for a substantial improvement of rehabilitation deliver, the way by which they determine restoration of function has not been yet clarified and the neurophysiological mechanisms underlying the recovery is still undefined. In a recent case series study (Chisari et al., 2014) the efficacy of Lokomat in gait rehabilitation and its capability to act on motor control has been tested in a group of stroke patients, resulting in a significant improvement after the training in clinical scales. Strength and Motor Unit firing rate of Vastus Medialis were also recorded and analyzed: no increase of force was observed whereas a significant increase of firing rate of Vastus Medialis was recorded, suggesting an effect of training on motorneuronal firing rate that may contribute to the improvement of motor control. In any case large effect size and robust effects of robotic treatment have not yet been fully demonstrated.

Concerning the upper extremity, impaired arm and hand function contributes considerably to limitations in the ability to perform activities of daily living (ADL). One of the goals of post stroke rehabilitation is to regain arm and hand function, since this is essential to perform ADL independently. Most of the robotic devices applied in clinical practice offer the possibility of choosing among four modalities for training: active, active-assisted, passive and resistive (Chang and Kim, 2013). These terms refer to subject’s status during interaction. In active mode, performance arises from subject contribution only, whereas in passive mode the movement is performed by the robot regardless of subject’s response. In active-assisted mode, the user performs active movement at the beginning and the robot acts only in particular conditions (i.e., if the target has not been reached in the requested time), systematically leading to success. Finally, resistive mode consists of resisting the movement received from the subject, so the robot makes the movement quite more difficult (Basteris et al., 2014). On this background, the “assistance-as-needed” control was conceived to encourage patients’ active motion. In this approach, the robotic device is able to either assist or correct the movements of the subject, with the aim to manage simultaneous activation of efferent motor outputs and afferent sensory inputs during training (Belda-Lois et al., 2011). Current assist-as-needed strategies aim to provide the suitable definition of the desired upper limb trajectory in space and time the robot must generate to assist the subject during the task (Frisoli et al., 2011).

Robotic devices for upper limb rehabilitation are also divided in end-effectors and exoskeletons. End effectors act by applying mechanical forces to the distal joints of the arm. An example of devices which act as end-effector is the “MIT Manus” (Lo et al., 2010). The advantage of end-effector devices is to guarantee an easy setup; the disadvantage is due to the lack of control of the proximal segments of the arm, and this could result in undesired, compensatory patterns of movement. Conversely, exoskeleton robotic devices are designed so that robot axes are aligned with anatomical segments of the subject’s arm. Exoskeletons allow direct control of individual joints, which can minimize abnormal posture or movement, but their construction is more complex and more expensive than that of the end-effectors. An example of exoskeleton device is the “ARMEO” (Taveggia et al., 2016).

For what concerns end-effectors, there are several studies comparing these devices with conventional therapy. A comparison between conventional therapy alone and robotic training combined with conventional therapy have been done in a sample of 56 subacute stroke patients (Fasoli et al., 2004), and the latter approach showed a greater improvement. This datum stands for a positive impact of end-effector devices on upper-limb recovery in patients in the subacute phase of stroke, thus recommending their use (Chang and Kim, 2013). Differently, another study conducted on a large sample of chronic stroke patients (Lo et al., 2010) showed that robot-assisted upper limb training with end-effector device and intensive conventional therapy determined the same degree of clinical improvement after 12 weeks of rehabilitation, although after further 24 weeks of treatment the robotic approach resulted in further improvement. Another study involving chronic patients (Hsieh et al., 2012) compared high-intensity robot-assisted training with control treatment group and low-intensity robot-assisted training with control treatment group and found significantly greater improvement after robotic training only when performed in the high-intensity modality. These insights indicate that the intensity is the most relevant parameter in the rehabilitation of upper limb with robotic devices in chronic stroke patients, when end-effector are used. An effect of robot-assisted therapy on ADL function is observed only in patients with subacute stroke (Chang and Kim, 2013).

As regards Exoskeleton-type robot devices for upper limb recovery, almost all trials performed up to now comprised patients in the chronic stage of stroke. Among them, one study reported a significantly better effect on spasticity in the robot-assisted therapy group than in the conventional therapy group (Fazekas et al., 2007). In contrast, ADL function improved more markedly in the conventional therapy group that received the same amount of treatment. Other reports demonstrate no significant difference between robot-assisted therapy with exoskeleton devices and conventional therapies (Kahn et al., 2006; Mayr et al., 2008; Housman et al., 2009). In addition, there are no randomized controlled trials that investigate robot-assisted therapy with exoskeleton devices in patients with subacute stroke. Therefore, at this time there is insufficient evidence to draw a definite assertion as to what is the real effectiveness of exoskeleton systems for the rehabilitation of upper limb after stroke.

**Figure 3:**
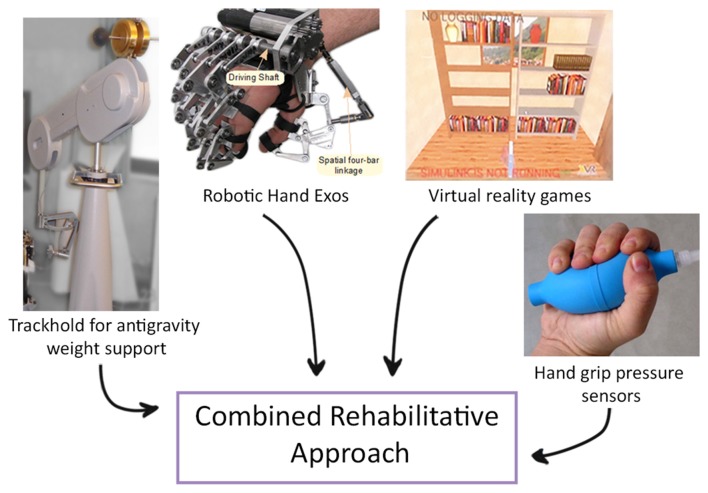
**Example of a novel robotic system that integrates functional grasping, active reaching arm training and bimanual tasks.** An example of a novel robotic system that integrates functional grasping, active reaching arm training and bimanual tasks, consisting of: (i) Virtual Reality: software applications composed of rehabilitative and evaluation tasks; (ii) TrackHold: robotic device to support the weight of the user’s limb during tasks execution; (iii) Robotic Hand Exos: active hand exoskeleton to assist grasping tasks; and (iv) Handgrip sensors to support the bilateral grasping training and evaluation (modified from Sgherri et al., 2017).

It has also been demonstrated that performing a task that mimics an ADL and that requires the coordination of more than one joint, a progressive complexity of the exercise and a greater number of DOF is more effective than standard two-dimensional reaching task (Schaefer and Hengge, 2016). Therefore, novel devices that combine the effect of end-effectors and exoskeletons have been proposed. One example is a new system that has been designed to combine functional grasping and active reaching in the same task; it also allows to perform bimanual exercises and consists of a system for weight support for the proximal joints, a robotic hand exoskeleton and a virtual reality interface (Figure 3; Barsotti et al., 2017). This integrated device showed improvement in motor performance and kinematic and strength metrics in a group of chronic stroke patients after an intensive rehabilitative treatment protocol (Sgherri et al., 2017).

To draw a conclusion, the impact of robot-assisted training is still debated: it guarantees intensive, repeatable, task-specific training, but up to now it seems an implementation to rather than a replacement for conventional physical therapy. Randomized controlled trials with large sample of patients are needed to draw more defined conclusions. A precise analysis of the economic burden and of the effectiveness of robotic rehabilitation is required.

Nevertheless, some authors argue that task-specific training alone is more likely to enhance behavioral compensation than effective recovery, highlighting the importance of providing the human equivalent of the “enriched environment” to augment the generalizing effect of spontaneous biological recovery instead of promoting compensation strategies (Zeiler and Krakauer, 2013). From this point of view, task-specific training is suggested to be delivered only involving ecologic and global tasks (i.e., reaching and grasping) and if accompanied by other approaches able to augment, prolong, or mimic the post-stroke sensitive period, such as NIBS techniques (see above, Section “Non-Invasive Modulation of Post-Stroke Plasticity in Humans”).

In this perspective, a better understanding of the central mechanisms underlying both spontaneous and training-guided recovery becomes mandatory in order to maximally take advantage from the brain capacity to reorganize its neural networks after damage. The neural mechanisms underlying the possible improvement led by robot-based therapeutic approach are still unclear, although recent studies have begun to shed light on this topic (Takahashi et al., 2008; Edwards, 2009; Posteraro et al., 2009; Pellegrino et al., 2012; Várkuti et al., 2013). An improved knowledge of these neural processes could help to ameliorate the effectiveness of the robotic therapy, and design combined protocols, i.e., robot-based and plasticizing therapy, able to boost the functional motor recovery. Further studies will allow a better understanding of the effect of rehabilitation on neural plasticity in order to adapt treatment resources to meet the needs of each patient and optimize the recovery process. In this context, animal models can be a suitable solution to investigate neural and motor changes after stroke and possible rehabilitative strategies, allowing the integration of behavioral, molecular and electrophysiological data.

### Robotic Devices for Rodents

Recently, some robotic devices designed for interaction with rodents, in particular rats, have been developed to study a particular task or training, demonstrating the successful integration of mechatronics and robotics with such animal models. The idea to exploit robotics in combination with animal models is not addressed to developing new devices for animals and then translating them to new systems for human, that already have reached a high level of technology and complexity. Rather, the robot developed to interact with animals tend to mimic human robot devices to try to investigate mechanisms at the basis of robot-based rehabilitation as well as providing quantitative, reliable and accurate data where qualitative and experimenter-biased tests are still intensively used.

Most of these studies have focused on the development of technological-advanced devices aimed at to assessing and training gait function of spinal cord injured (SCI) rodents.

Reinkensmeyer’ group (Nessler et al., 2005) proposed the first example of exoskeleton for rodent, the *Rat-Stepper*, a device consisting of two lightweight robotic arms connected to the rat hindlimbs, a body weight support system (BWS), and a motorized treadmill. This device can deliver precise amounts of weight support as well as perturbing and assisting the injured animals during the stepping. The authors claimed that the Rat-Stepper was able to accurately and easily capture step trajectories, provide sensitive and valid measures (e.g., length of the step or velocity) of locomotor recovery following contusion injury and even discriminate among different SCI levels (Nessler et al., 2006). This capacity to quantify the animal motor performance, without being affected by the experimenter’s bias, makes robotic devices extremely appealing to the researchers. Moreover the same group designed a smaller but similar device for mice, the *Mice-Stepper*, used to provide an assisted-as-needed (AAN) paradigm for rehabilitate SCI animals. The results showed the highest level of recovery in AAN trained mice, measured through the number, reliability and frequency of steps during the testing sessions (Cai et al., 2006). The Mouse-Stepper was also used in combination with serotonin agonist, quipazine, and demonstrated significant recovery of locomotor function in SCI mice (Fong et al., 2005).

Another example of robotic apparatus specifically-targeted to research into the field of SCI is the *IronRat* system, recently developed in the laboratories of the MIT (Song and Hogan, 2015). The IronRat permits the rats to move freely in an open space (i.e., arena) and perform a wide range of voluntary movements. It is composed of a BWS, a monitoring-control system and the *Rat Backpack*. This latter component was designed as an exoskeletal module for the rat’s hindlimbs, mounted on the lower back of the animal and coupled with hindlimb ankle joints. It allows hindlimbs to have two actuated DOF along the sagittal plane of ankle motion, and provides force feedback with the animal’s hindlimb to compensate friction not compromising the back-driveability. Although this system appears quite bulky, after an initial habituation, healthy animals attached to the active Rat Backpack performed locomotion with stride length, stride duration, and duty cycle analogous to unconstrained overground movements (evaluated with BBB Locomotor Rating Scale; Basso et al., 1995). The IronRat is a promising tool in the field of SCI research, even though it has been tested only in healthy animals so far.

In the treatment of SCI on human, a key point is that the therapists can optimally adapt training to the requirements of each patient based on their own expertise and the severity of the injury. However both Rat-Stepper and IronRat cannot replicate the degree of adaptation of human therapists because of the limits imposed by their mechanical constraints. Florez et al. (2016) tried to overcome this issue by developing an exoskeleton for rats equipped with soft pneumatic actuators (SPA) attached to the hindlimbs. This system is composed of a BWS, a body structure customized to fit onto different sized subjects and an active part moved by the SPA. SPA are made in polymeric elastomers, can be tailored to any specific embodiment (Holland et al., 2014), are highly compliant and can produce high power-to-weight ratios (over 10 N with a 100 g actuator; Florez et al., 2016). The softness introduced by this actuation method makes the device suitable for interaction with fragile environments, as animal body, and also control fine mechanical stimulation that should allow SCI rats to produce a broad range of foot trajectories, improving the physical interaction between rodent and robot, as shown in their preliminary experiments.

A device that have been already proficiently validated and tested in the SCI treatment is the robotic exoskeletons developed by the Courtine’ group (Dominici et al., 2012; van den Brand et al., 2012). Dominici et al. (2012) designed a versatile robotic neuroprosthesis consisting of three translational axes frame (*x*, *y*, *z*), as well as one rotational axis (φ). This device is provided with a suspension support (i.e., BWS) and a multidirectional elastic decoupling system that allows high-fidelity force control in each of the four DOF of the structure. The authors tested the efficacy of this device in combination with epidural electrical stimulation and tailored cocktail of serotonine and dopamine agonists (Dominici et al., 2012; van den Brand et al., 2012). To enable stepping in complete SCI rats: their results showed the capacity of this system to assess pattern generation and dynamic equilibrium, as well as promoting advanced locomotor capacities as walking and even stair climbing in SCI rats. This robotic postural interface was used to force, the rats to actively use their paralyzed hindlimbs in order to locomote bipedally (van den Brand et al., 2012) by encouraging an active participation of the animal.

Although so far most of the robot-based devices proposed for rodents have been developed to investigate gait function after SCI in rats, some robotic systems have been designed also to train and study forelimb function. These systems generally tend to automate already existing systems as lever pulling or similar. One of the first example of sensorized system to evaluate forelimb performance was presented by Fowler et al. (1994) where a simple force-sensing operandum was used to study the effect of a specific drug during a continuous pressure task performed by healthy rats. This system was thus used as means to quantify the performance in an unbiased-way.

Francis and Chapin (2004) developed a 1 DOF lever arm for rats. It was implemented to investigate feedforward and feedback control mechanisms in rodent forelimb motor tasks: indeed animals were trained to grasp the end-operandum and move it to some fixed targets. Different force field perturbations, implemented following different paradigms (i.e., viscous, constant torque, spring and isometric force fields) were opposed to the animal movement but, as author claimed, animals were able to adapt themselves to face all of these paradigms.

The application of force fields to effect on forelimb movements was studied and implemented also by Gassert’s group who developed a robotic platform, *ETH Pattus*, designed to be used in motor learning experiments with rats (Vigaru et al., 2013). This compact device is highly transparent, has 3 DOF manipulandum (consisting of a pantograph frame provided with a further rotational DOF) and is capable to provide forces up to 2 N to guide or perturb rat forelimb movements, in accordance to different and possible force field implementations. Their preliminary experiments with healthy rats showed that ETH Pattus is able to collect data and quantitatively describe the dynamic interaction with the animal’s paw. Despite its potentiality as a valuable tool to assess recovery after brain lesions (e.g., stroke), it was initially thought to investigate planar reaching and pronosupination movements, such as required when performing skilled reaching movements.

Skilled reaching is a natural behavior in rodents and its modeling has currently assumed a great importance and interest. In fact, although rodents display behavioral specializations quite different from humans, skilled reaching shares many similarities with the homologous behavior in humans. For example, velocity profiles in rat movements (Vigaru et al., 2013) exhibited bell-shaped profiles similar to humans (Morasso, 1981). However, traditional analyses assess motor performance using end-point measures of success/failure and only recently more sophisticated kinematic measures of movement execution (Dominici et al., 2012; Lai et al., 2015) or assessment based on mechatronic devices have been carried out.

Hays et al. (2013) developed an automatic system to train rats to perform a isometric pull task, described as novel technique to precisely measure the strength and the function of the forelimb during a skilled reaching task. The animals were taught to reach for a handle linked to a stationary force transducer and pull it isometrically until getting a threshold force level followed by the delivery of a food reward. Force, success rate, pull attempts as well as latency to maximal force are all quantitative parameters monitored by this device, through the commercial *MotoTrak* software (Vulintus, USA). Moreover, the authors tested the capacity of this system to detect deficits in rats undergone an ischemic stroke in M1 in comparison with other traditional tests (i.e., pasta matrix and end-points skilled reaching): the animals showed evident impairments in performing the task as described by all the three tests but only the isometric pull task was sensitive enough to detect significant deficit by 6 week post-lesion (Sloan et al., 2015). The same group from the UT Dallas also designed and tested a similar device for mice and observed long-term impairments in pulled force (Becker et al., 2016).

Similarly to the Hays’s work, Reinkensmeyer’s group recently developed another robotic interface, the Robotic Rehabilitator of the Rodent Upper Extremity (RUE), This 1 DOF system allows rats to voluntarily perform a task of reaching followed by the pulling of a bar in order to retrieve a food reward (Sharp et al., 2016). The bar is connected to an interface by means of which the force required to accomplish the pulling task can be changed, simply varying the stiffness of a voice coil actuator used to generate the resistance force. The authors used the RUE as a tool to assess the upper limb force production of healthy rats in comparison with a well-known method for measuring strength, i.e., the Grip Strength Meter (GSM; Anderson et al., 2004). They observed that the rats performed a pulling force higher compared to the maximum strength exerted through the GSM, showing that a more advanced system as the RUE can unveil more detailed behavioral information (e.g., rat’s capacity to perform higher force). To date, RUE has been used as assessment tool but its utility even as rehabilitation device in SCI and stroke models seem to be clear.

**Figure 4:**
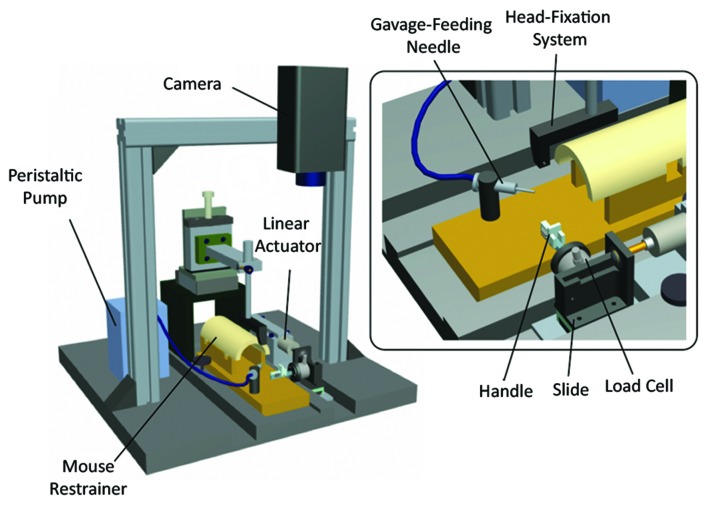
**M-Platform for training and measuring motor performance of mice forelimb.** Schematic of the robotic device. The main components are indicated: head fixation system, peristaltic pump with a gavage-feeding needle for liquid reward delivery, restrainer, linear actuator, camera to record forelimb position, plastic handle for the retraction task, linear slide and load cell for forces detection. Modified from Spalletti et al. (2014).

Indeed robotic devices could be used to investigate the robot-mediated recovery after neural injury and try to boost recovery. In this context, mouse models could seem more appealing for the possibility to exploit advanced techniques (e.g., optogenetics, knock-out technology) in combination with motor training. However, in spite of the advanced several methods and models, to date there is an evident lack of robotic systems to train mouse and in particular the functionality of its forelimbs. Spalletti et al. (2014) proposed the *M-Platform* (Figure 4), a 1 DOF robotic device for mice that mimics a human robot system for upper limb stroke rehabilitation (Arm-Guide; Reinkensmeyer et al., 2000). Head-restrained mice can be trained to perform intensive and highly repeatable exercises by retracting their forelimb previously extended by a linear actuator. Forces exerted during the task, time required for task execution (t-target), number of submovements and attempts (i.e., force peaks not overcoming a static friction force) can be quantified for each trial. The M-Platform is able to detect motor deficit in ischemic mice (i.e., local damage in M1) and to train animals to reach pre-injury performance. Moreover this system is currently being combined with advanced techniques, such as optogenetics and mesoscale brain imaging, to study plasticity mechanisms after stroke as well as pharmacological treatments aimed to boost recovery.

In conclusion, these devices try to implement similar control strategies or to extract the measures used in clinical cases, as trajectories, velocities and forces, with the obvious limitations related to the model and the task (Vigaru et al., 2013). However, these first examples of devices for robot-mediated neurorehabilitation in rodent models are paving the way to increase the understanding of the mechanisms underlying clinical improvements in patients affected by neural diseases (Grimaldi and Manto, 2013).

## Conclusions

In this review article, we analyzed recent advances in the understanding of the mechanisms of post-stroke network reorganization and discussed the use of the state-of-the-art therapeutic techniques, such as NIBS and robot-based protocols.

It is now well known that an ischemic damage leads to spontaneous neuroplasticity in perilesional tissue, promoting map reorganization observable both in human and animal models. Many researchers have focused on the study of different neurotransmitter systems which have an important role in this remapping process, such as the glutamatergic and the GABAergic networks. In fact, whereas pharmacological activation of AMPA receptors improves motor outcomes after stroke, a reduced inhibition is correlated to an enhanced plasticity. After stroke, an augmented activity in the contralesional hemisphere has been also reported in patients and animal models, particularly in acute stage, and even the contralesional neuronal connections appear to be altered. However the role of the healthy hemisphere in recovery is still controversial and debated.

A reasonable hypothesis is related to the lesion volume and the amount of spared tissue: in the case of sufficiently small injuries allowing the reorganization of spared adjacent motor areas, the contralesional hemisphere activity would have a negative impact on the recovery process. Indeed, subjects recovering from stroke showed changes in interhemispheric influences between the two hemispheres, probably due to a decrease of the ipsilesional neuronal activity and an increase of the contralesional one. For this reason, many studies have tried to shed light on the dynamics of the coupled brain areas and the local modifications of inhibitory and excitatory neurotransmission after stroke. A loss of sensorimotor function has been correlated to deterioration of inter-hemispheric functional connectivity in animals. In human strokes, a loss of coherence and connectivity between the hemispheres has been documented, that slowly recovers in parallel with spontaneous behavioral improvements.

In such a scenario, NIBS can have the potential to foster recovery. Indeed several studies observed improvements in motor deficits after exciting the affected hemisphere or inhibiting the healthy one by implementing “top-down” protocols of rTMS or tDCS. Although a general consensus about the best protocols has been not achieved yet, it is believed that the general “state-dependency” is a critical feature of the cortical neural processing and it plays a crucial role on the efficacy of NIBS protocols.

Another important approach we discussed to promote network plasticity and functional recovery is the robot-based rehabilitation. These devices guarantee intensive, repeatable, task-specific training, but up to now they represent just an adjunct to rather than a replacement for conventional rehabilitation therapy. Robot-assisted therapy is successful on improving upper limb motor function in stroke patients and the possibility of delivering high intensity treatment is one of the most important features of robotic technologies. However there are insufficient evidences to draw a definite conclusion regarding its effectiveness. The possibility to study plastic mechanisms of functional recovery has recently led to the introduction of robot-based paradigms even in animal models, in particular rodents. Many groups have demonstrated the successful integration of robotics with such animal models and laid the foundation to study neural mechanisms at the basis of robot-based rehabilitation as well as providing quantitative and accurate information about the recovery after neural injury.

## Author Contributions

All authors contributed to writing and discussion of this review article.

## Funding

We gratefully acknowledge financial support from Fondazione Pisa (Project 158/2011). Work in the authors’ laboratory is also supported by Regione Toscana (RONDA Project, “Programma Attuativo Regionale” financed by FAS—now FSC) and ERC Advanced Grant “BrainBit”.

## Conflict of Interest Statement

The authors declare that the research was conducted in the absence of any commercial or financial relationships that could be construed as a potential conflict of interest.
